# A randomized controlled trial of meditation and health education on carotid intima-media thickness and major adverse cardiovascular events in Black men and women

**DOI:** 10.3389/fmed.2025.1513699

**Published:** 2025-03-19

**Authors:** Keith C. Norris, John Salerno, C. Noel Bairey Merz, Vidya Kaushik, Simon Gelleta, Amparo Castillo, Sanford Nidich, Carolyn Gaylord-King, Robert H. Schneider

**Affiliations:** ^1^Department of General Internal Medicine and Health Services Research, David Geffen School of Medicine, University of California, Los Angeles, Los Angeles, CA, United States; ^2^Formerly Department of Internal Medicine, College of Medicine, Charles R. Drew University of Science and Medicine, Los Angeles, CA, United States; ^3^Institute for Prevention Research, Fairfield, IA, United States; ^4^Barbara Streisand Women’s Heart Center, Smidt Heart Institute, Cedars-Sinai Medical Center, Los Angeles, CA, United States; ^5^Department of Public Health, Des Moines University, Des Moines, IA, United States; ^6^Community Health Sciences, Department of Public Health, University of Illinois, Chicago, IL, United States; ^7^Center for Natural Medicine and Prevention, College of Integrative Medicine, Maharishi International University, Fairfield, IA, United States

**Keywords:** meditation, stress management, Transcendental Meditation, health disparities, minority health, cardiovascular disease

## Abstract

**Introduction:**

Black Americans suffer from disproportionately high rates of cardiovascular disease (CVD). Psychosocial stress contributes to this disparity. Previous studies reported that the Transcendental Meditation (TM) technique reduced CVD risk factors, surrogate endpoints, and clinical events in high-risk populations. However, no study has evaluated the effects of stress reduction with meditation on surrogate CVD markers such as carotid intima-media thickness (cIMT) along with CVD clinical events. Therefore, this randomized clinical trial evaluated the long-term effects of meditation and health education (HE) on cIMT and CVD events in high-risk Black adults.

**Materials and methods:**

Participants were Black women and men with CVD or at high risk who were randomized to either TM or HE. The primary outcome was a change in cIMT measured using B-mode ultrasound at baseline and 12 months. The main secondary outcome was major adverse cardiovascular events (MACE) at 5 years (maximum) of follow-up. Other secondary outcomes were MACE at 1 and 10 years of follow-up, blood pressure, and serum lipids after 1 year. Exploratory variables were a comparison of cIMT changes to historical controls and MACE after 14 years.

**Results:**

There were 197 randomized participants, of whom 136 completed posttest for cIMT. After 1 year, the TM and HE groups showed average cIMT changes of −0.0004 and −0.0003 mm, respectively, with no significant difference between the groups. Additionally, there were no significant differences between the groups in lipid levels or BP. However, both TM and HE groups showed prevention of progression of cIMT compared to historical controls at 12 months. In the survival analysis of MACE, there was a 65% relative risk reduction in the TM group after 5 (maximum) years of follow-up (HR = 0.346; 95% CI = 0.134–0.893; *p* = 0.017). At 1 and 10 years of follow-up, there were significant risk reductions in the TM vs. HE group, which was not significant at 14 years (all yearly maximums).

**Discussion:**

Both treatment groups demonstrated prevention of progression of cIMT over 12 months compared to historical controls. However, the TM group showed a relative risk reduction for MACE of 65% at 5 years. Therefore, as a lifestyle modification method, TM may be useful in the secondary prevention of CVD in this and possibly other high-risk groups.

**Clinical trial registration:**

ClinicalTrials.gov, NCT05642936.

## Introduction

Among ethnic groups in the United States, Black men and women suffer from disproportionately high rates of atherosclerotic cardiovascular disease (ASCVD) morbidity and mortality (1–3). Growing evidence indicates that this disparity in CVD may be caused, at least in part, by excessive psychosocial and environmental stress (4–6).

Substantial evidence indicates that social determinants of health, notably psychosocial stress, are a major contributor to the onset and progression of CVD (7, 8). Underlying mechanisms include vascular inflammation (9), oxidative stress (10), and endothelial nitric oxide inhibition (11). It has been demonstrated that the attributable risk associated with psychosocial stress factors across diverse populations is similar to traditional CVD risk factors (12). A recent review found that traditional risk factors (e.g., hypertension, metabolic syndrome, and dyslipidemia) may emanate in part from the physiological effects of psychosocial stress factors (13). The American Heart Association Scientific Statement on the Mind-Heart-Body Connection (14) and the European Society of Cardiology Guidelines on Cardiovascular Disease Prevention in Clinical Practice (15) recognized the contributions of psychosocial stress and the potential of stress-reducing interventions to prevent CVD in high-risk groups.

While there have been studies on a variety of behavioral interventions in the primary and secondary prevention of CVD, randomized controlled trials of stress reduction employing the Transcendental Meditation (TM) technique showed decreases in CVD risk factors, surrogate endpoints, and clinical events in high-risk Black Americans and the general population (16–21). In an earlier feasibility and efficacy trial, Castillo-Richmond et al. reported significant regression in carotid intima-media thickness (cIMT), a surrogate marker for CVD, in a cohort of Black men and women with hypertension participating in the TM program compared to health education (HE) for an average of 7 months (22).

Considering the demand for research on preventing health disparities associated with ethnicity and social determinants of health (23), we undertook this study with a long-term analysis to evaluate the effects of meditation compared to conventional health education on a surrogate marker of ASCVD, namely carotid intima-media thickness, over 1 year, as well as major adverse cardiovascular events over the same 1 year period and in the long term over several years.

## Methods

### Design

This was a randomized controlled single-blinded community-based clinical trial. The study enrolled Black men and women participants at risk for ASCVD. All participants provided written informed consent after approval from their primary care physicians. After baseline testing, participants were randomly assigned to either a stress reduction program with the TM technique or a HE program consistent with AHA/ACC guidelines for lifestyle prevention of CVD (24). The HE condition controlled for expectancy, attention from the instructor, protocol adherence, and other non-specific factors. The intervention period was 12 months. The follow-up period for cIMT, CVD risk factors, and fatal and non-fatal MACE was 12 months and, for fatal MACE, a maximum of 14 years after randomization. All participants continued with usual care from their community medical care providers.

The clinical center for the trial was King-Drew Medical Center in Los Angeles, CA. Participants were recruited primarily from the angiography units of King-Drew and nearby hospitals. The core laboratory for cIMT analysis was Cedars-Sinai Medical Center in Los Angeles, California, and the administrative and data coordinating center was the Institute for Natural Medicine and Prevention in Fairfield, Iowa. The study of cIMT and MACE was conducted from October 2000 to December 2014. Due to a lack of availability of resources and personnel after the initial funding period, data analysis was later completed in April 2024.

### Participants

Participants were recruited from the cardiac catheterization laboratory and cardiologist referrals at King-Drew Medical Center along with radio and newspaper advertising and local community organizations (senior centers and churches). The inclusion criteria were as follows: ethnicity: African American, self-identified, age: 21 years or older, sex: male or female; CVD: history of either diabetes, myocardial infarction, coronary artery bypass grafting (CABG), percutaneous coronary intervention (PCI), coronary angiography indicating at least one vessel with >50% stenosis, and/or carotid artery intima-media thickness (cIMT) ≥ 0.72 mm for men and ≥ 0.65 mm for women.

Participants were excluded if they had either a recent myocardial infarction, unstable angina, CABG, PCI, or stroke (within the previous 3 months), or a carotid artery endarterectomy, arrhythmia—atrial fibrillation, a second or third degree AV block, heart failure class III or IV or ejection fraction less than 30%, clinically significant valvular heart disease, hepatic or renal failure, major psychiatric disorder (psychosis, schizophrenia, bipolar), current alcohol or other drug abuse dependency disorder, non-cardiac life-threatening illness, or were participating in a formal stress management program.

Participants were tested at baseline over three separate clinic visits all within a 2-week baseline testing period. The primary outcome of carotid IMT was measured at baseline and 12 months. Testing for the secondary outcomes of blood pressure, cholesterol, exercise, and substance use was collected at baseline, 3 months, and 12 months posttest. Adverse clinical events were monitored at each scheduled clinic visit (i.e., 3 and 12 months). Fatal MACE were determined from National Death Index records from 2000 to 2014.

### Randomization and blinding

After completion of baseline data collection, participants were randomly assigned to one of the two study conditions, TM or HE with stratification by gender, age, mean arterial pressure, and LDL cholesterol. Allocation concealment was carried out by a biostatistician who performed the computerized randomizations according to protocol and then contacted the project manager who, in turn, directly contacted the study participants to notify them of their treatment group. All investigators, data collectors, and other clinic staff were masked to treatment assignment, except the project manager who did not collect data from participants. Primary care physicians were also masked to the treatment assignments of their patients. Since participants were necessarily aware of their treatment status, this behavioral intervention trial was a single-blind, randomized controlled design.

### Outcomes

The primary outcome was carotid artery intima-media thickness (cIMT) change over 12 months. Carotid IMT, measured by B-mode ultrasound, has been validated as a reliable surrogate marker for ASCVD (25). Carotid IMT measurement offers advantages for tracking atherosclerosis for several reasons: It is non-invasive, assesses the early stages of atherosclerosis, provides a continuous, precise measurement, and thus requires a smaller sample size than other markers. Carotid artery wall thickness has been found to be a risk factor and a valid surrogate endpoint for CVD clinical events, including myocardial infarction and stroke (25–27).

The main secondary outcome was MACE after 5 years (maximum) of follow-up. Other secondary outcomes were MACE at 1 and 10 years (maximum) of follow-up, blood pressure, and serum lipids changes after 1 year. Exploratory outcomes included changes in cIMT compared to historical controls and MACE at 14 years.

#### cIMT testing protocol and data acquisition

Intima-media thickness (IMT) of the common carotid artery was non-invasively measured by quantitative B-mode ultrasound. The B-mode carotid ultrasonography was performed using a Toshiba system with high resolution, a TOMTEC Image Analysis System, and a state-of-the-art real-time scanner. The carotid examination was performed with participants in a supine position with the head rotated to the left side (28). Intima-media thickness is the distance between interfaces 2 and 3 (near wall) and 4 and 5 (far wall) on the longitudinal view. The distal 1 cm of the right common carotid artery was examined. The primary outcome measure was the average of near and far wall maximum IMT measurements at the right distal common carotid artery. Frozen images at the site of maximal wall thickening or stenosis in anterior and lateral positions and a short segment of real-time scanning with continuous ECG were recorded. For further analysis of wall thickness and % luminal stenosis, the images were transferred to a computer system where they were digitized and stored on disk. A fully automated, computerized, edge-detection tracking method was used to minimize variability and increase statistical power with a small sample size (29). This method, which can detect early stages of atherosclerosis, is considered more precise than manually traced measurements and reduces variability (28–30).

#### cIMT image analysis

Images of the carotid wall were digitized on a video frame grabber and installed on a PC computer. Studies were identified by a study code and blindly examined. These images were analyzed using a Data Translation DT 2862, an image processing board, and a Prosound system. This software used an edge-detection algorithm that enabled automatic detecting, tracking, and recording of the intima/lumen surfaces and media/adventitia surfaces.

The analyst identified three to six points of the blood–intima boundary; then, the program fitted a smooth curve to these points as a guide for edge tracking; a computer search was conducted along paths perpendicular to the curve for conditional edge points of maximal intensity change; then, the gradient value for each conditional edge was compared to the maximum gradient of all conditional edge points, and those with gradients less than 20% of the maximum were eliminated.

This process was repeated for the media–adventitia boundary. IMT in the distal right common carotid was computed as the average distance, over 120 boundary points (representing 1 cm of the carotid wall), between the far wall lumen-intima boundary and the far wall media–adventitia boundary. The distance between characteristic echoes arising from blood–intima interface and media–adventitia interface was the combined IMT measure. The same reader evaluated both baseline and follow-up scans. The reproducibility of the analysis was assessed yearly by rereading 10% of the exams.

#### Secondary outcomes

Blood pressure was measured at 0, 3, and 12 months by a trained technician three times using a manual sphygmomanometer after instructing the participant to sit quietly for 5 min without practicing any formal relaxation technique. Three readings were taken 1 min apart and then averaged for each of the three separate clinic visits. To minimize the potential effect of white coat hypertension, the study’s BP reading was based on the average of the last two clinic visits only. Blood was drawn for lipid panel measurement after 12-h fasting and stored frozen for later analysis.

Study staff surveyed participants during their follow-up clinic visits (at months 3 and 12) to assess adverse clinical events since their previous visit. This included collecting information on the nature, type, and severity of the event, whether hospitalization occurred, and the length of stay. Major adverse cardiovascular events (MACE) were collected and defined as death from CVD or hospitalization due to myocardial infarction, unstable angina, coronary revascularization, stroke, or heart failure. All non-fatal MACE were determined from hospital records and adjudicated by blinded investigators. For participants who did not return during the intervention period and all participants thereafter, we assessed fatal MACE from the National Death Index (NDI) (National Center for Health Statistics, Research Triangle Park, North Carolina). The NDI provides an estimated accuracy of 93–98% in identifying the vital status of participants (31, 32). The database of enrolled participants’ records was searched against the National Death Index database beginning in November 2000 through 31 December 2014. The NDI search included all participants who were randomized, regardless of whether they completed the 1-year posttesting. Causes of death were obtained from the National Death Index Plus service, which provided coded causes of death based on the *International Classification of Diseases*, Ninth Revision. Cause-specific mortality was based on the underlying cause of death. Codes 390–459 were defined as death from diseases of the circulatory system, including all cardiovascular diseases.

### Interventions

The TM program was used as a mind–body intervention for its effects on physiological correlates of stress and related CVD outcomes and because of its standardization, reproducibility, and validity (21, 33–36). TM is a meditation technique derived from the Yoga tradition that has been adapted for contemporary practice (35–37). Since its adaptation, over ten million people worldwide and three million in the US have learned this technique through standardized courses (35–37). TM is described as a simple, natural technique practiced for 20 min twice a day while sitting comfortably with the eyes closed (36, 37). It is reported that the ordinary thinking process settles down, leading to a distinctive wakeful hypometabolic state characterized by neural coherence and physiological rest (38–42).

Standardized teaching materials and formats were used (36, 37). The TM program was taught in a standard seven-step course of instruction consisting of six 1.5–2 h individual and group meetings taught by an instructor certified by the Maharishi Foundation USA (36). Thereafter, follow-up and maintenance meetings were held weekly for the first month, biweekly for the next 2 months, and monthly thereafter for the remainder of the 12-month study period.

The active control intervention was a cardiovascular health education program (HE) designed to match the format of the experimental intervention for instructional time, instructor attention, participant expectancy, social support, and other non-specific factors (43). The content was based on American College of Cardiology and American Heart Association guidelines (24, 44, 45). The instructors were professional health educators. To match the 20-min allocated time at home for the TM group, the HE participants were advised to spend at least 20 min a day at home practicing heart-healthy behaviors, e.g., exercise, healthy meal preparation, and non-specific relaxation. Care was taken to separate both intervention groups regarding class time and location to minimize contact and communication.

The experimental and control groups had the same number of group meetings (40 over the 12-month study period), and meeting attendance was collected for both groups. Both groups recorded the regularity of home practice during clinic visits throughout the intervention period. In addition, the TM group used a home practice log card for their participants to track their treatment compliance on a daily basis. In parallel, the HE group filled out a form that asked about the time spent applying the knowledge gained in class to their home life (e.g., exercise and practicing healthy cooking).

### Data analysis

The sample size was determined using statistical power analysis (46), based on cIMT as the primary outcome. The projected effect of the interventions was based on the pilot study by Castillo-Richmond, et al. (22) which found a net reduction of 0.15 mm (with a standard deviation of 0.30 mm) in cIMT for the TM group relative to the health education group, yielding an operational effect size (mean change/SD of change) of 0.50. Based upon an analysis of covariance (ANCOVA) on change from pretest to posttest, two-tailed tests at the 0.05 level, 80% statistical power, and 30% attrition over 12 months, power analysis indicated that 92 subjects would be required per group. Demographic and baseline variables were compared between the two groups using a two-sample *t*-test or Wilcoxon rank-sum test for continuous variables and Pearson’s chi-square test for categorical variables.

The statistical analysis for the cIMT included all study participants randomized to either TM or health education who had baseline and 1-year follow-up cIMT data, that is, by modified intent-to-treat analysis (47). The effect of TM compared to health education on cIMT change was calculated using ANCOVA with baseline carotid IMT as the covariate. Secondary analyses examined the effect of risk variables as a treatment effect moderator using a two-way analysis of variance (ANOVA) that included treatment group, risk factor variable, and treatment*risk interaction effect in the model adjusted for baseline cIMT as a covariate. In addition, covariates were identified from the demographic and baseline variables that were found to be significantly associated with cIMT change. These variables were then included as covariates in the ANCOVA model for testing the effect of TM vs. health education on cIMT change.

Furthermore, we conducted *post-hoc* analyses comparing the TM and HE interventions to historical controls. This comparison is based on recommendations by Marion and Althouse (48), FDA regulatory guidance (49), and European Medicines Agency scientific guidelines on the choice of control group in clinical trials (50). The historical external control for cIMT change was taken from Bots et al.’s systematic review and meta-analysis of 23 randomized placebo-controlled trials involving 2,693 control subjects, conducted during the same approximate time period as the current trial (51). We compared the effects of the two intervention groups, TM and HE, to the changes in the placebo groups from the 2003 systematic review and meta-analysis by Bots et al. Carotid IMT progression rates in the TM and HE groups were compared to each other and to the historical controls using ANOVA. Exploratory analyses were conducted on high-risk subgroups to assess changes in cIMT.

As described above, during the first year of intervention and follow-up, non-fatal MACE were collected during participant visits and verified by hospital records. Fatal MACE were collected from the National Death Index. The survival data were analyzed using the Kaplan–Meier and Gehan-Breslow-Wilcoxon tests, following the intention to treat principle. The Gehan-Breslow-Wilcoxon test was used to compare survival distributions between the groups in the trial, as it is sensitive to early differences in survival times and does not rely on the proportional hazards assumption, making it a robust choice for our dataset with a high density of early events and censored observations (52). Survival analyses were conducted at 1, 5, and 10 years, with an exploratory analysis at 14 years. The 1-year analysis was conducted as it marked the end of the intervention and in-person follow-up period. The 5-year analysis served as the main secondary outcome, selected for its alignment with the average follow-up duration in randomized controlled trials (RCTs) on cIMT progression, as highlighted in a recent systematic review and meta-analysis. The 10-year analysis provided an additional long-term assessment of outcomes. The 14-year analysis was exploratory, representing the maximum follow-up period for study participants.

## Results

### Baseline characteristics

**Figure 1:**
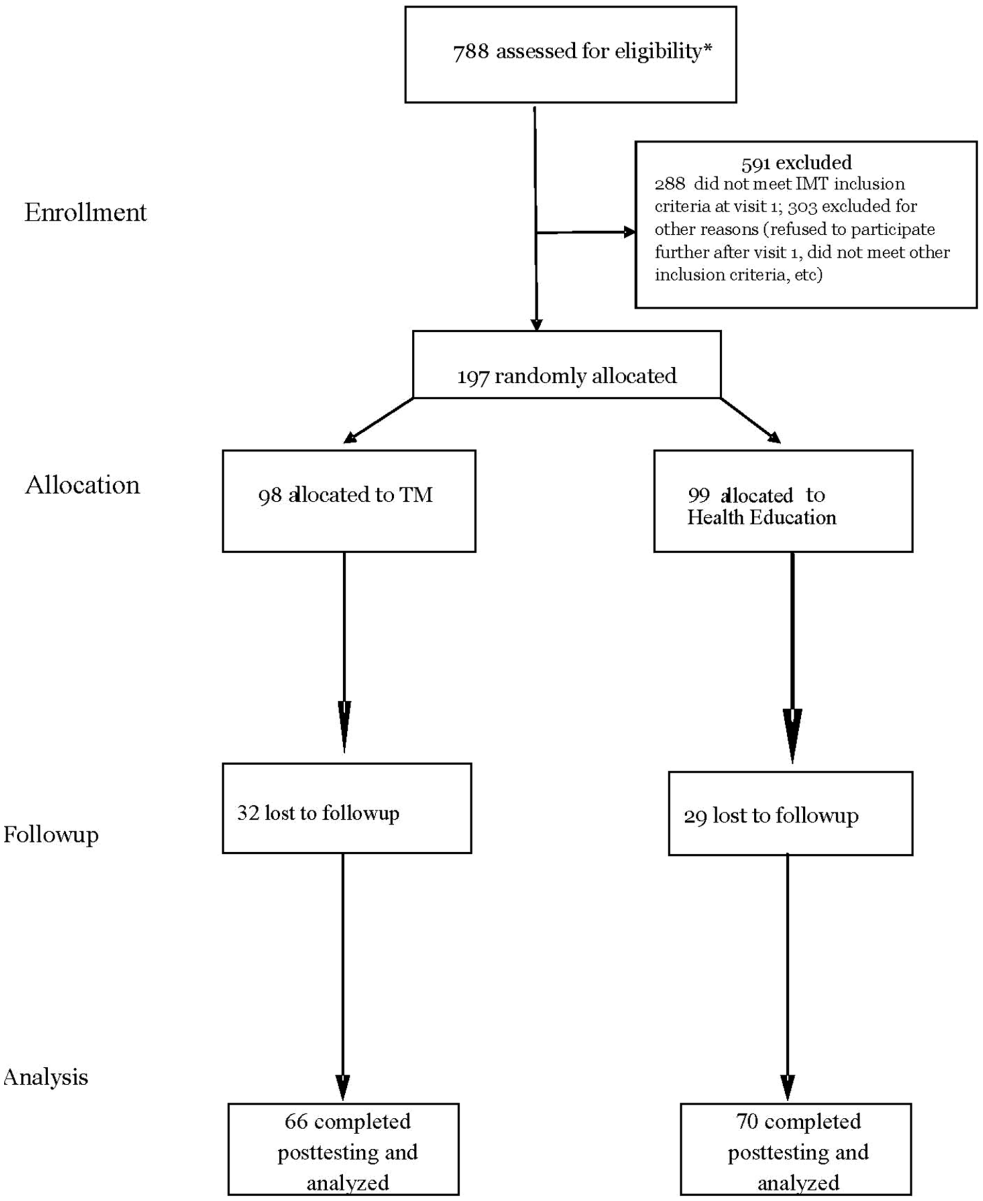
Participant flow diagram. *indicates approximate number of subjects eligible for study.

Of the 197 randomized study participants, 142 had a 1-year follow-up, of which 136 had posttest cIMT measurements. Figure 1 shows the patient flow diagram according to CONSORT guidelines for reporting parallel group randomized trials (51).

Table 1 shows the baseline characteristics and demographics for each treatment group. The groups were generally similar; 44% were women; mean age was 57 years. There were no significant baseline differences between the groups.

**Table 1:** Comparison of treatment groups on demographic and baseline variables.

Variable	TM group Mean	HE group Mean	TM group SD	HE group SD
Sex (% female)	61.2%	59.6%		
Age, yrs	55.4	54.9	11.0	10.1
Married (%)	24.7%	25.5%		
Years of education	13.2	13.2	2.1	2.3
Income				
<$10,000	49.0%	44.3%		
$10,000–20,000	22.9%	23.7%		
$20,000–50,000	21.9%	19.6%		
>$50,000	6.3%	12.4%		
Weight (kg)	94.5	95.6	23.6	22.8
Body mass index, kg/m^2^	33.2	33.2	7.0	7.0
Carotid intima-media thickness (mm)	0.886	0.901	0.174	0.214
Systolic blood pressure, mm Hg	136.5	135.1	20.1	18.7
Diastolic blood pressure, mm Hg	75.6	76.2	10.8	10.6
Mean arterial pressure, mm Hg	95.9	95.8	12.4	12.2
Heart rate, beats/min	71.9	75.5	10.8	10.3
Diabetes	66.2%	68.6%		
Previous myocardial infarction	28.6%	21.2%		
Smoking	23.5%	21.2%		
Alcohol (drinks/week)	1.4%	0.7%	3.8	2.5

Medications	TM Group %	HE Group %
Diuretic	33.7	42.4
Calcium channel blocker	46.5	43.4
Antiarrhythmic	7.1	10.1
ACEI	48.0	56.7
ARB	8.2	5.1
Beta blocker	21.4	16.2
Aspirin	28.6	29.3
Statin	32	33
Hypoglycemic	65.3	61.6

TM, Transcendental Meditation; HE, health education; SD, standard deviation.

### Primary analyses of cIMT

**Figure 2:**
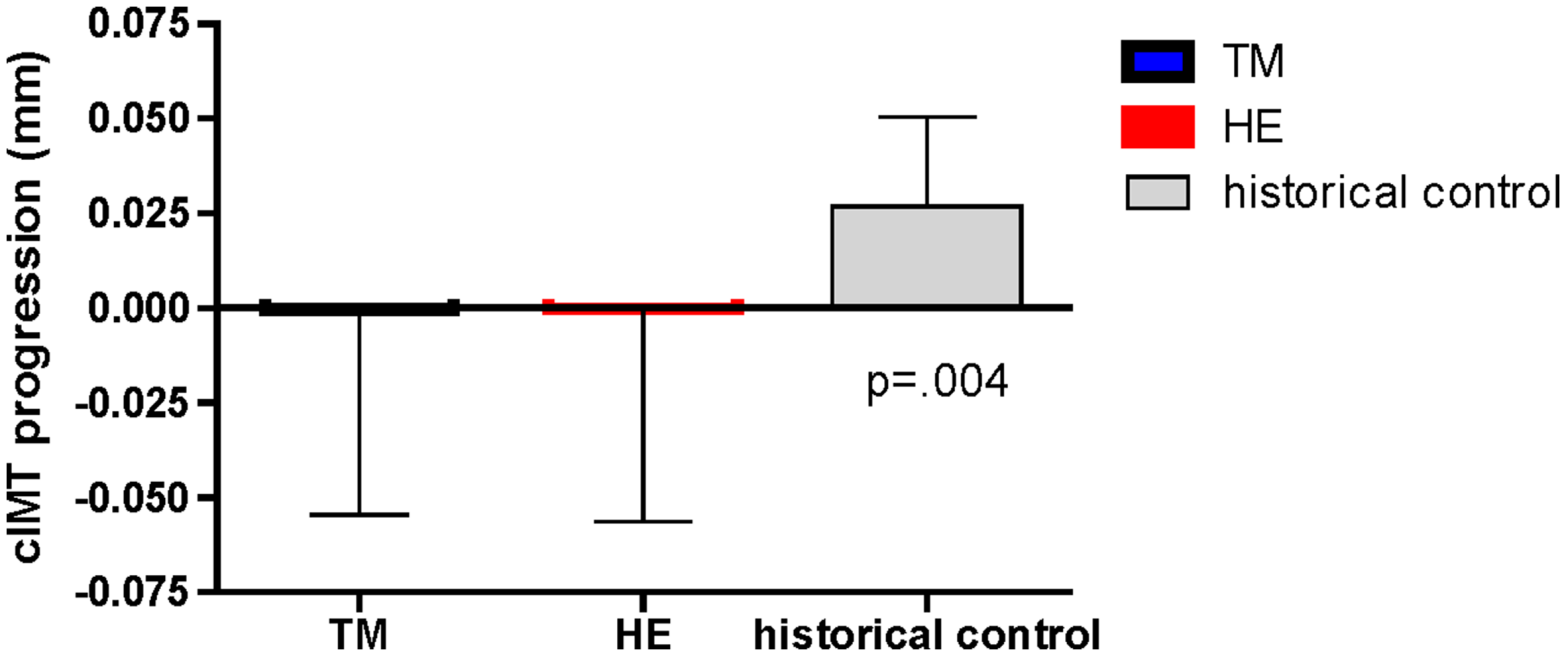
Effects of the Transcendental Meditation and health education groups compared to historical controls on carotid intima-media thickness progression over 1 year.

Posttest completion for the primary outcome of cIMT at 1 year was 72%. Among the sample of 136 subjects with pre- and posttest cIMT, the TM group showed a reduction of −0.0004 mm, and the HE group showed a reduction of −0.0003 mm. When the mean changes of the three groups (TM, HE, historical controls) were compared by one-way ANOVA, there was a significant difference in progression of cIMT (*p* = 0.004) (Figure 2).

### Subgroup analysis of cIMT

**Figure 3:**
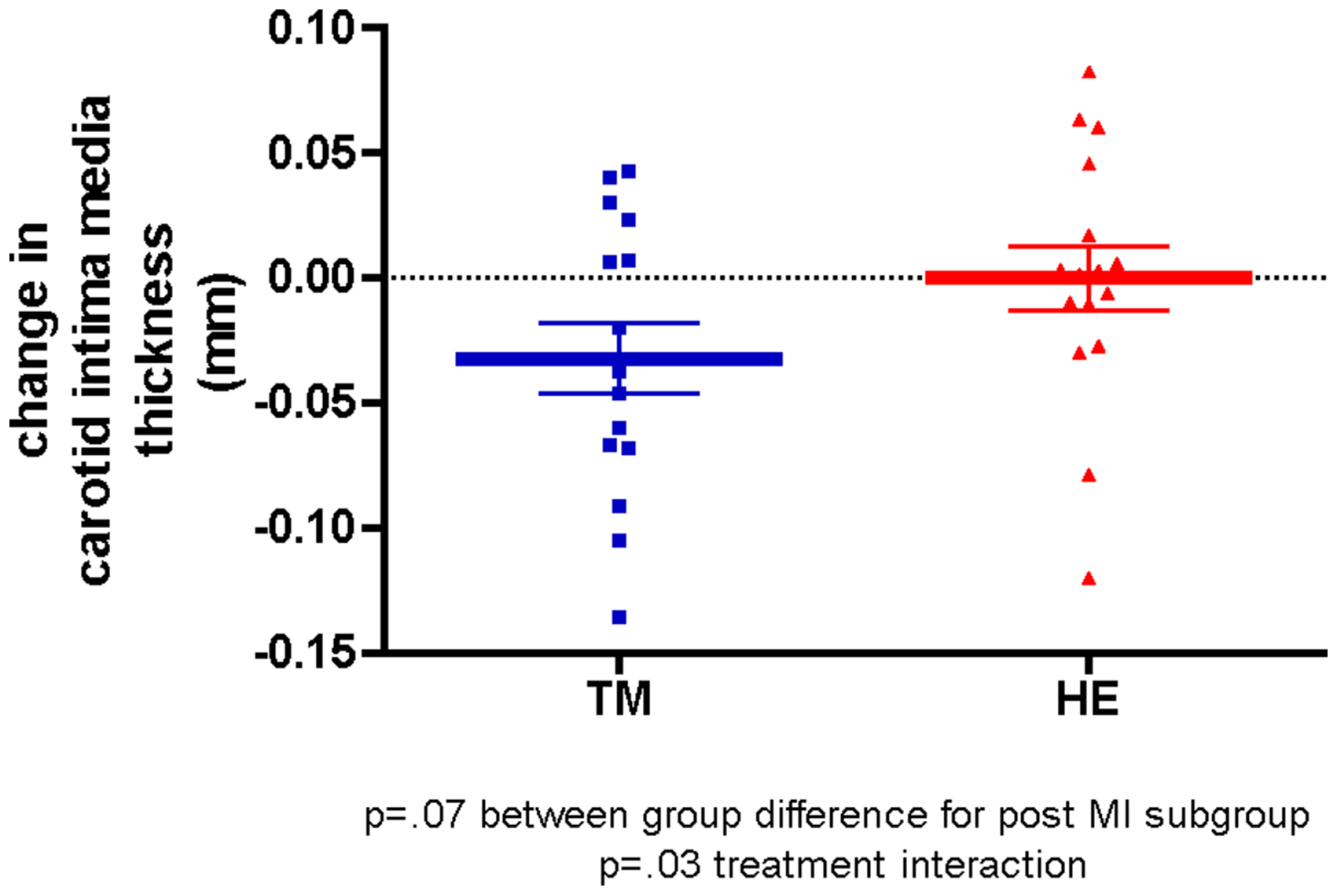
Adjusted mean changes in carotid intima-media thickness in the post-myocardial infarction subgroup over 1 year.

Exploratory subgroup analyses conducted using a two-way ANOVA to assess whether a risk factor moderated the effect of treatment suggested that post-MI status was a significant moderator of treatment effect, after adjusting for covariates (*p* = 0.029). The analysis showed a difference in treatment effect between participants with and without previous MI, with a favorable trend for the effect in TM vs. HE subjects among those with previous MI (*p* = 0.07) (Figure 3).

### Risk factor analyses

Systolic blood pressure fell in both the TM and HE groups. There were no significant between-group differences in changes in blood pressure, cholesterol, or exercise. However, the HE group had lower triglyceride levels compared to the TM group at posttest (*p* = 0.004).

### Survival analyses

Table 2 shows the hazard ratios (HR), 95% CI, and significance of the TM vs. HE groups with 1-, 5-, and 10-year survival analyses. It also includes the mean follow-up periods and Ns for each analysis time point. Table 3 shows the breakdown and distribution of MACE in each treatment group. The TM group showed relative risk reductions in non-fatal and fatal MACE at year 1 and a relative risk reduction in fatal MACE at 5 and 10 years compared to the HE group. At 5 years, the main survival time point there was a 65% risk reduction (*p* = 0.017) in TM vs. HE groups. Figure 4 shows the Kaplan–Meier survival curves to 10 years. In the 14-year exploratory analysis, the between-group difference of 15 MACE in the TM group vs. 22 in the HE group was not significant (HR = 0.68; CI = 0.35–1.31; *p* = 0.15).

**Table 2:** Major adverse cardiovascular events (MACE) at each timepoint for the Transcendental Meditation group compared to the health education group.

	1 year	5 years	10 years
Hazard ratio	0.071	0.346	0.485
CI (95%)	0.009–0.542	0.134–0.893	0.226–1.044
*p*-value	*p* = 0.0007	*p* = 0.0172	*p* = 0.0435
Mean follow-up time (years)	1.07	4.61	8.62
Remaining N CVD event free	185	167	157

N = number of subjects.

**Table 3:** Major adverse cardiovascular events (MACE) by treatment group.

	# events	# events	# events
Endpoint	TM	HE	Both
CVD mortality	10	19	29
Non-fatal MI	0	0	0
Non-fatal stroke	0	0	0
Coronary revascularization	0	3	3
Heart failure hospitalization	0	3	3
Angina/CHD hospitalization	2	1	3
Other CVD hospitalization	0	2	2
TOTAL	12	28	40

**Figure 4:**
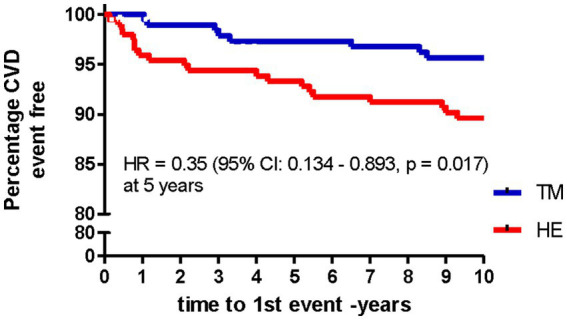
Major adverse cardiovascular events (MACE) in Transcendental Meditation (TM) and health education (HE) groups over 10 years (*n* = 197).

### Adherence

Treatment adherence, as indicated by meeting attendance over the 12-month study period, was 65% for the TM group and 71% for the HE group. Home practice adherence over the 12-month study period for the TM group was 70%, with participants considered regular (i.e., practicing at least once a day), based on forms administered at the clinic posttest after 12 months. For the self-reported home practice logs, the TM patients reported 87% regularity after the 12-month study period. For the HE group, at-home practice compliance, as reported on the administered clinic forms, was 84% regularity over the 12-month study period.

## Discussion

In this randomized controlled trial of Black women and men at risk for CVD who were allocated to either the TM program or a health education program (HE) for 12 months, both treatment arms showed modest regression of cIMT (−0.0004 and −0.0003 mm/yr for TM and HE, respectively). This contrasts with historical controls who demonstrated significant progression of cIMT over the same time period (average + 0.0147 mm/yr) (48). Thus, the two behavioral interventions in this trial may be considered to have prevented the progression of cIMT. Corresponding to this change, during the 12-month intervention period, there was a 93% relative risk reduction in composite MACE for the TM group compared to the HE group. Analyses of long-term follow-up for survival showed significant relative risk reductions of 65% at 5 years and 51% at 10 years for MACE for TM vs. HE. Thus, it appears that the effect of TM may be sustainable in terms of survival up to at least 10 years. Factors that may have limited or played a role include lower long-term compliance with the practice, and/or maintaining other healthier lifestyle changes in the TM group that attenuated over time.

Based on the suggestions from previous studies that participants with more severe CVD would respond more to intervention (53), subgroup analyses stratified by baseline characteristics were conducted for cIMT. This analysis suggested that post-MI status was a significant moderator of treatment effect on change in cIMT (*p* = 0.029), with a favorable trend (*p* = 0.07) for greater regression of cIMT (−0.033 mm/yr) among TM participants compared to HE participants in the post-MI group. A similar pattern of regression was observed in other subgroups with more severe diseases, such as CVD, hypertension, and diabetes, although these results did not reach statistical significance (data not shown).

As to the clinical significance of the prevention of progression of cIMT in the present trial, Willeit et al. conducted a meta-analysis of cIMT thickness and CVD risk comprising 119 clinical trials with 100,667 patient participants over an average follow-up of 3.7 years using a meta-regression approach (24). This large meta-analysis estimated that interventions reducing cIMT progression by 0.01 mm per year would yield a relative risk of 0.84 (75–0.93) for MACE (fatal plus non-fatal). Furthermore, prevention of 0.02 mm progression was associated with a relative risk for CVD of 0.76 (0.67–0.85). The results were similar for pharmacologic and non-pharmacologic trials, time of conduct, different types of cIMT measurement, and other factors. Therefore, applying these parameters, the 0.015 mm reduction in progression in the intervention group effect in the present trial might be anticipated to yield a relative risk of 0.80 or, in other words, a risk reduction of 20% for CVD events over nearly 4 years. The TM group showed a greater relative risk reduction of 65% compared to HE, which may be due to the limited sample size or larger effect of the TM intervention due to other mechanisms in addition to cIMT prevention of progression. Notably, a systematic review by Khanal et al. suggested that mechanisms of sympathetic nervous system and hypothalamic–pituitary–adrenal axis activation, inflammation, oxidative stress, insulin resistance, and psychosocial stress in addition to traditional CVD risk factors may contribute to reduced risk for morbidity and mortality in TM practicing subjects compared to controls (20).

The present results are consistent with an earlier pilot trial of TM and HE by Castillo-Richmond et al. (22). In this randomized controlled trial, 60 Black men and women with hypertension were studied for changes in cIMT before and after 7 months of TM or health education intervention. In this study, there was a −0.15 mm IMT significant reduction observed in the TM group relative to health education controls. Both studies evaluated subjects of similar ethnicity (Black), age (i.e., mid-50s), and sex (60% women). Although it confirmed the direction, the present trial did not confirm the magnitude of the earlier treatment effect. Differences between the findings of the earlier pilot and current trial may be due to differences in baseline cIMT values. The average cIMT at baseline for the Castillo-Richmond pilot study was 1.6 mm and for the present phase II trial was 0.86 mm (22). Alternatively, differences in cIMT ultrasound methods may have contributed to the variation in the magnitude of results (54). We found more modest reductions in cIMT relative to proportionally larger reductions in MACE in the TM group vs. HE. However, earlier TM studies in patients at high CVD risk showed reduced intermediate markers of CVD, that is, left-ventricular hypertrophy (51), insulin resistance (18), and myocardial ischemia (55). Thus, it is possible that some or all of these markers not measured in this study may have contributed to reduced CVD mortality in the TM group.

There also appeared to be no differences in 1-year medication changes between the groups that could account for the group differences in long-term mortality (see Table 1). Alternatively, possible differences in long-term home practice compliance (i.e., 5–10 years) in the TM group relative to the HE group may have contributed to the larger reduction of MACE relative to cIMT, BP, and lipids in this study. It should also be noted that the TM subjects had no-cost benefits including lifetime support of TM counseling (i.e., checking) by a certified teacher as well as monthly advanced lectures at their nearest TM center. The HE subjects, on the other hand, were on their own after the 1-year study without support from health educators.

The 5-year survival in the present study was consistent with a long-term randomized controlled trial (RCT), which showed significantly higher survival in the TM group compared to a health education program at 5.4-year average follow-up for Black men and women with coronary heart disease (17). Another long-term investigation that pooled two RCTs of 202 Black and Caucasian adults with high BP and followed over 7.6 years found a 23% relative reduction in all-cause mortality and a 30% relative risk reduction in cardiovascular mortality in the TM group compared to behavioral controls (19).

For comparison, several pharmacologic and lifestyle modification interventions for the prevention of CVD and progression of cIMT have been reported (25, 52). For example, a multimodality intervention study, which was a pooled analysis of two trials, investigated the combined effect of TM, a healthy diet, herbal supplements, and yoga exercises in older patients at high risk for CVD. The results showed that after 9–12 months of intervention, a significant cIMT reduction (−0.17 mm) was observed in the experimental group compared to an enhanced and active health education group (56).

There are plausible physiological mechanisms for preventing the progression of cIMT and reducing rates of MACE in this trial. These include attenuation of downstream changes in physiological correlates of stress, such as sympathetic nervous system overactivation, hypothalamic–pituitary–adrenal dysfunction, pro-coagulation, increased inflammation and oxidative stress, among others. These are known to contribute to pathophysiological pathways of endothelial damage and dysfunction, hypertension, atherosclerosis, thromboembolism, myocardial infarction, stroke, and death (21, 57–60). A unified systems medicine model of CVD and meditation is presented elsewhere (33).

### Limitations

One limitation of this clinical trial is the use of historical controls. Marion and Althouse recommend comparing with historical controls when untreated control groups are not practical. They also elaborate on the limitations of this approach due to possible methodological differences in the control vs. experimental study conditions (48). While there were differences in timing and participants in our study, a meta-analysis of 119 RCTs suggests that time of study intervention, time and type ultrasound measurement, availability of individual patient data, primary vs. secondary prevention trials, and participation of female participants do not significantly modify intervention effects on cIMT progression and their relations to CVD risk (25).

CIMT progression may be accelerated in Black women and men in association with excess CVD in this population, driven by high exposure to both interpersonal and structural ethnic discrimination, as well as adverse social determinants of health (2, 3, 5, 61, 62). However, there is a limitation in generalizing the present findings to other ethnic populations. The current sample comprised Black women and men, who, as a group, receive suboptimal treatment across most, if not all, health and health-related domains due to historical and ongoing discriminatory policies and practices, which have contributed to their placement in a low US ethnic caste status (63). This could lead to a differential response compared to other socially defined ethnic groups. However, other studies on Transcendental Meditation in diverse populations, particularly white women and men, have shown improvement in cIMT and other CVD surrogate and clinical endpoints that are consistent with the present findings (18, 19, 55). Fiscal constraints on posttesting, high residential mobility, and scheduling conflicts after 1 year may have been some of the reasons for the attrition rate of 28%. Although the attrition rate may appear to compromise the generalizability of our findings, attritors and completers were generally similar in baseline characteristics. Both the TM group (30% attrition) and the health education group (26% attrition) were equally affected by attrition. Moreover, 30% attrition, although high, was considered in the power analysis and sample size calculation.

Due to HIPAA constraints and other privacy restrictions on participants who departed the study, medical records to track and adjudicate non-fatal CVD events could not be obtained for the subsequent years after the 1-year study period. Therefore, only MACE data related to CVD mortality could be obtained through the National Death Index of the US Dept. of Health and Human Services.

The subgroup analysis was exploratory, yet hypothesis-driven as described above. Cautions associated with reporting extended follow-up in cardiovascular clinical trials have been elaborated by Gaudino et al. (64). Another possible contributing factor to the non-significant between-group differences in cIMT may be that the cutoff entry criteria were set too low (65). Finally, while two-dimensional ultrasound imaging of cIMT has been demonstrated to be valid and reliable (25, 53), it has been suggested that newer ultrasound measures, e.g., three-dimensional vascular and ultrasound imaging (VUS), have been introduced, allowing more accurate quantification of more diverse multi-territorial atherosclerosis (66, 67). Future studies might investigate total plaque changes in meditation and lifestyle modification interventions using these higher dimensional, multi-territorial vascular imaging techniques to confirm and extend these study findings (68).

## Conclusion

This trial evaluated the efficacy of a stress reduction meditation method, the Transcendental Meditation technique, compared to a health education program on cIMT and MACE in Black women and men at risk for CVD. Both lifestyle modification interventions prevented the progression of cIMT compared with historical controls. However, there was a significant relative risk reduction of MACE after 5 years of 65% in the TM group compared to HE. This pattern was seen at 1 year and extended out to 10 years and attenuated at 14 years. These findings, taken together with the results of other randomized controlled trials on meditation, suggest that the application of Transcendental Meditation as a lifestyle modification may be useful in the primary and secondary prevention of CVD in high-risk populations.

## Data Availability

The raw data supporting the conclusions of this article will be made available by the authors, without undue reservation.
